# Physical flow effects can dictate plankton population dynamics

**DOI:** 10.1098/rsif.2019.0247

**Published:** 2019-08-07

**Authors:** J. R. Woodward, J. W. Pitchford, M. A. Bees

**Affiliations:** 1 Department of Mathematics, University of York, York, UK; 2 Department of Biology, University of York, York, UK

**Keywords:** inertia, cell density, ecological dynamics, harmful algal blooms

## Abstract

Oceanic flows do not necessarily mix planktonic species. Differences in individual organisms’ physical and hydrodynamic properties can cause changes in drift normal to the mean flow, leading to segregation between species. This physically driven heterogeneity may have important consequences at the scale of population dynamics. Here, we describe how one form of physical forcing, circulating flows with different inertia effects between phytoplankton and zooplankton, can dramatically alter excitable plankton bloom dynamics. This may impact our understanding of the initiation and development of harmful algal blooms (HABs), which have significant negative ecological and socio-economic consequences. We study this system in detail, providing spatio-temporal dynamics for particular scenarios and summarizing large-scale behaviour via spatially averaged bifurcation diagrams. The key message is that, across a large range of parameter values, fluid flow can induce plankton blooms and mean-field population dynamics that are distinct from those predicted for well-mixed systems. The implications for oceanic population dynamic studies are manifest: we argue that the formation of HABs will depend strongly on the physical and biological state of the ecosystem, and that local increases in zooplankton heterogeneity are likely to precede phytoplankton blooms.

## 1. Introduction

It is commonplace to assume that the principal effect of fluid flow on an oceanic ecosystem is to mix biological populations and the nutrients that they rely on. Indeed, such mechanisms lie at the heart of our understanding of annual cycles in primary productivity, whereby seasonal interactions between an upper photic mixed layer and deeper nutrient-rich waters can cause rapid increases in algal biomass over a few weeks [1]. It is natural, then, to question whether oceanic flows have significant effects upon the population dynamics, either quantitatively or qualitatively, particularly in the absence of gradients in nutrient or light or detailed behavioural responses. Is it reasonable to assume that fluid circulation ensures an essentially well-mixed environment over a range of ecologically meaningful length and time scales?

While there is much work on individual zooplankton–phytoplankton interactions in shear flows [2,3] and many observations of plankton heterogeneity associated with large-scale currents [4], there is little consensus about the impact of general flows on population dynamics. A traditional view is that flows and associated effects should either wholly mix the biology or separate the biology into distinct well-mixed patches (e.g. in circulating flow structures), each with a full complement of interacting species [5]. However, simulation evidence [6] suggests that turbulence can actively drive small-scale patchiness for motile phytoplankton and experimental evidence in Palma Bay in the Balearic Islands finds a causative relation between plankton size structure and slowly varying annual flow features [7]. Here, we show that physical effects can disaggregate foodweb components and that this effective segregation can in principle dictate large-scale ecological dynamics.

**Figure 1.**
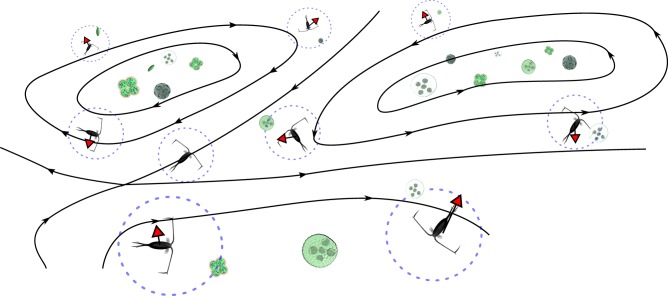
Sketch illustrating inertial drift (red arrows) of zooplankton (black) out of eddies, allowing phytoplankton (green) within the eddies to escape grazing control by zooplankton. The responsive radius of each zooplankton is illustrated by a dashed circle. Flow streamlines are given by black lines and arrows.

Planktonic organisms have different physical characteristics from the fluid in which they are found. For example, they have different densities, sizes and shapes [8]. As a result, different species will experience different drift relative to the surrounding fluid (figure 1); particle trajectories will not match streamlines of fluid flow; and inertia and sedimentation drive potentially complex trajectories [9,10]. The magnitude of these effects has to be carefully assessed, but it is clear that even a small amount of drift perpendicular to streamlines in regions of high shear can lead to very large dispersion in the direction of the flow (the well-known Taylor dispersion [11]). Non-swimming organisms do not simply follow fluid flow streamlines; depending on their relative density and shape they can accumulate or spend more time in regions of high shear or vorticity [12] or in flow regions collinear with gravity [13] that naturally arise in marine flows.

If predator and prey species have different densities, or differences in size and shape, then these variable inertial characteristics will lead to them being transported differently by the flow. This can, in principle, have ecological consequences. For example, Reigada *et al.* [12] demonstrated that spatial segregation of predators and prey driven by inertial effects in synthetic turbulence can allow a local prey population to grow in a relatively unconstrained manner. The predator population responds at the fringes of the burgeoning prey population; the local predator population increases, the original inertial flux is countered by a diffusive flux and, eventually, the predators consume a large part of the prey population. In this way, the requirement present in many existing mathematical descriptions of bloom phenomena (for example, [14]) for a large external perturbation to kick-start the system away from dynamic equilibrium is superfluous.

Here, we build on Reigada *et al.* [12] and investigate the effects of inertia on advected excitable phytoplankton–zooplankton dynamics for a simple two-dimensional cellular flow. This flow is chosen because it incorporates streamline curvature, and can represent an array of eddies, while the relevant flow magnitude and length and time scales can be transparently controlled. We, systematically, investigate a range of physical flow scales and ecological parameters. Further to this, we explore the spatially averaged bifurcation structure from regions of parameter space that impart non-bloom dynamics through to regions with ecologically realistic stationary or oscillatory bloom behaviour. We discover that small amounts of inertia can not only kick-start algal bloom formation in a circulating flow, but that as a consequence they can drastically alter phytoplankton–zooplankton interactions and thus mean population dynamics. Our results suggest that studies of phytoplankton–zooplankton dynamics that assume that turbulence simply mixes species in small regions may not tell the whole story.

In contrast to seasonal ocean-scale cycles in productivity, harmful algal blooms (HABs) occur on a smaller spatial scale and are difficult to predict. Nevertheless, these major biological events lead directly to extensive ecological and socio-economic damage on a global scale. They occur when local algal populations undergo a period of rapid growth, causing toxic or damaging effects to surrounding ecosystems. HABs have been shown to have widespread health impacts on fish and shellfish [15], marine mammals [16], birds [17] and humans [18]. HAB events are happening more often and in more places than ever before [19], and they particularly endanger small communities in the developing world that depend on a healthy catch of seafood to sustain the local population.

With HABs having such increasing negative impacts on public health and the worldwide economy, it is of growing importance that we discover and fully understand the mechanisms by which they may be triggered. Here, we argue that the temporal and spatial scales associated with simple oceanic flow features, combined with realistic physiological differences between phytoplankton and zooplankton, are likely to be important drivers of HAB dynamics.

## 2. Methods

We follow Reigada *et al.* [12] in constructing a model for the trajectories of plankton species subject to inertial effects. Font-Muñoz *et al.* [7] provide experimental evidence that size structure is directly affected by kilometre-scale flow structures over a yearly cycle in a real-world coastal system. They conclude that inertial effects alone can account for the observed heterogeneity.

For a spherical particle at position **X** with velocity **V** in a non-stationary fluid velocity field **U(X, t**), the equation of motion for the particle determined by Maxey & Riley [9] is given by

2.1 $$\begin{array}{rl} m_{\;p}\frac{dV}{dt} & =m_{\;f}\frac{DU(X)}{Dt}+6\pi a\eta (U(X)-V)\\ & \;-\frac{m_{\;f}}{2}\left(\frac{dV}{dt}-\frac{dU(X)}{dt}\right)\\ & \;-6\pi a^{2}\eta \int_{-\infty }^{t}\frac{d(V-U(X))/d\tau }{(\pi \nu {\left(t-\tau )\right)}^{1/2}}\;d\tau , \end{array}$$

where *m*_*p*_ is the particle mass and *m*_*f*_ is the mass of fluid displaced by the particle, *η* and *ν* are the dynamic and kinematic viscosity of the surrounding fluid, respectively, and *a* is the radius of the particle. Terms on the right-hand side account for the Bernoulli force from the undisturbed flow, the Stokes viscous drag, an added mass effect and the Basset history force (see [9] for details). It is clear that consideration of Kolmogorov scales may become important for larger plankton in the open ocean, and the strict validity of equation (2.1) is open to question in this range (see [20]). However, we avoid these complexities and exploit the leading-order drift of particles across streamlines in larger rotating flows. The main aim in our study is to provide a simple model of species segregation due to an inertial effect in a well-defined flow. Reducing the size of the plankton will reduce the rate of drift, but will not change the qualitative dynamics.

We simplify significantly by making approximations proposed by Taylor [21], Auton *et al.* [22] and Druzhinin & Ostrovsky [23] (see [10]) to equation (2.1), which yields an equation for velocity that depends only upon the position of the particles. This enables us to define an effective (non-dimensional) particle velocity field

2.2 $$V(r)=U(r)+\frac{R-1}{A}[U(r)\cdot \nabla ]U(r)+O(A^{-2}),$$

where **U(r)** is the ambient fluid velocity field, *R* = 3*m*_*f*_/(2*m*_*p*_ + *m*_*f*_) is the (non-dimensional) Bernoulli number describing a ratio of masses, and *A* = 12*πaη*/(2*m*_*p*_ + *m*_*f*_) is the reciprocal of the characteristic viscous drag time of the particle. The Stokes number St is given by a ratio of 1/*A* and the characteristic flow time scale, such that $\text{St}=u_{0}/l_{0}A$, where *u*_0_ and *l*_0_ are flow velocity and length scales, respectively. (To derive (2.2), St is considered small, the approximate form of (2.1) is integrated and exponential transients are neglected.) Note that Font-Muñoz *et al.* [7] contains a typographical error on the left-hand side of their governing equation (2.1), but the simulation results remain accurate (I Tuval 2019, private communication).

Following Reigada *et al.* [10], the divergence of (2.2) can be written in terms of the magnitude of the local strain-rate *S* and vorticity *Ω* of the original flow **U**. Hence,

2.3 $$\nabla \cdot V=\frac{R-1}{A}\left(2S^{2}-\frac{{\left|\Omega \right|}^{2}}{2}\right).$$

Therefore, particles move across streamlines and tend to aggregate in regions of negative divergence. If the organism is more dense than the fluid, (*R* < 1), then accumulation is expected in regions where *S*^2^ > |*Ω*|^2^/4, meaning there is high strain and low vorticity, while less dense organisms, (*R* > 1), accumulate in regions of low strain and high vorticity (inside eddies). Neutrally buoyant particles (*R* = 1) are passively advected by the ambient flow and do not accumulate in any particular region (unless other terms in (2.1) are retained). Equation (2.2) provides the leading-order effect of inertia in a relatively simple Eulerian flow field.

The above approach is of real practical use as it allows us to consider population dynamics with a spatially continuous description across large length scales of interest (approx. 100 m). Note that in moving from a description describing individual organisms to a continuum, we must consider length scales much larger than the distance between organisms.

To achieve this, we construct a system of reaction–advection–diffusion equations of the form

2.4 $$\frac{\partial P}{\partial t}=-\nabla \cdot (V_{P}P-D_{P}\nabla P)+f_{P}(P,Z)$$

and

2.5 $$\frac{\partial Z}{\partial t}=-\nabla \cdot (V_{Z}Z-D_{Z}\nabla Z)+f_{Z}(P,Z),$$

where **V**_*P*_ and **V**_*Z*_ are the effective velocity fields for the phytoplankton and zooplankton, respectively, and *D*_*P*_ and *D*_*Z*_ are the diffusivity coefficients. Typically, we set *D*_*P*_ and *D*_*Z*_ equal as effective eddy diffusivity is likely to be significantly larger than that due to swimming.

The choice of excitable plankton dynamics is inspired by the general Truscott & Brindley [14] model of plankton blooms in a well-mixed system. The model considers two interacting trophic levels, phytoplankton (*P*) and zooplankton (*Z*). It consists of two nonlinear ordinary differential equations for *P* and *Z*. The model exhibits excitable dynamics: small perturbations return to the non-trivial steady state whereas larger perturbations can instigate a large excursion around phase space over an extended period, corresponding to a bloom. We therefore use the reaction terms *f*_*P*_(*P*, *Z*) and *f*_*Z*_(*P*, *Z*) from the Truscott & Brindley [14] model, such that

2.6 $$f_{P}(P,Z)=rP\;\left(1-\frac{P}{K}\right)-R_{m}Z\frac{P^{2}}{\kappa^{2}+P^{2}}$$

and

2.7 $$f_{Z}(P,Z)=\epsilon R_{m}Z\frac{P^{2}}{\kappa^{2}+P^{2}}-\mu Z,$$

where *r* is the maximum growth rate of phytoplankton, *K* is the phytoplankton carrying capacity, *R*_*m*_ is the maximum specific predation rate, *κ* is the half-saturation constant, *ε* is the efficiency of the zooplankton and *μ* is the linear death rate of the zooplankton.

There are three steady states of the system: a trivial equilibrium at the origin, zooplankton extinction at (*P*, *Z*) = (*K*, 0) and a coexistence state at

2.8 $$(P,Z)=\left(\frac{\kappa }{\zeta },\frac{r\kappa \epsilon }{\mu \zeta }\left(1-\frac{\kappa }{K\zeta }\right)\right),$$

where $\zeta =\sqrt{(\epsilon R_{m}/\mu -1}),$ meaning that coexistence is not possible unless *εR*_*m*_ > *μ*. At *ζ* = *κ*/*K*, the coexistence state collides with the zooplankton extinction state and the system undergoes a transcritical bifurcation. The trivial equilibrium is a saddle point of the dynamical system, while the zooplankton extinction point is a stable node when *ζ* < *κ*/*K* and a saddle point otherwise.

Following Truscott & Brindley [14], we find that the points at which Hopf bifurcations occur for the coexistence equilibrium are determined by the solutions to the cubic equation

2.9 $$K\zeta^{3}-K\zeta +2\kappa =0.$$

Descartes’ rule of signs tells us that there must be one negative and two positive real roots. However, by definition, *ζ* cannot be negative, and so there are two Hopf bifurcations. As *ζ* increases from 0, the first bifurcation causes a stable limit cycle to come into existence, and the second results in the coexistence equilibrium regaining linear stability as a stable spiral.

The effective velocity fields **V**_*ξ*_, *ξ* = *P*, *Z*, are given by

2.10 $$V_{\xi }=U+\beta_{\xi }[U\cdot \nabla ]U,$$

where *β*_*ξ*_, *ξ* = *P*, *Z*, are Maxey–Riley coefficients, with *β*_*ξ*_ < 0 for negatively buoyant particles and *β*_*ξ*_ > 0 for positively buoyant particles.

Reigada *et al.* [12] used a turbulent stationary flow as a background flow. We shall instead employ a (stationary) cellular flow [24]. This gives us some advantages as it allows us to have full control over the length and flow speed scales of the eddies in our model, meaning we can vary the maximum flow speed as a bifurcation parameter. This enables us to perform a full bifurcational study of the spatial averages of the two species with respect to both physical and ecological processes, and leads to a simplified one-dimensional system.

Here, we shall consider the simplest case where inertial effects become relevant for two-dimensional flow in a horizontal plane. However, for a vertical plane one must also include sedimentation and the Lambert–Beer law for light attenuation and thus growth dependent on light absorption by other organisms above a given position in space. Hence, the ambient fluid velocity **U** = (*U*_1_, *U*_2_) is given by

2.11 $$U_{1}=U_{0}\sin\left(\frac{2\pi x}{L}\right)\;\cos\;\left(\frac{2\pi y}{L}\right)$$

and

2.12 $$U_{2}=-U_{0}\cos\left(\frac{2\pi x}{L}\right)\sin\left(\frac{2\pi y}{L}\right)\;$$

for (*x*, *y*) ∈ [0, *L*]^2^, where *U*_0_ is the maximum speed of the flow and *L* is the diameter of a single circulatory cell, which can be written in terms of the streamfunction *ψ* = (*U*_0_ *L*/2*π*)sin(2*πx*/*L*)sin(2*πy*/*L*) and so satisfies incompressibility.

Typically, submesoscale eddies of horizontal diameter 0.1–10 km (smaller than large mesoscale eddies, 10–200 km) and vertical extent 0.01–1 km can persist in the ocean for days, with some submesoscale coherent vortices even persisting for years [25]. Constrained regions can also contain circulating flows; Font-Muñoz *et al.* [7] indicate in their study that they observed flow features with length scales of the order of a kilometre that were relatively stable and switched around annually.

Statistical measures of spatial features of the plankton are necessary to be able to compare the different spatial distributions resulting from various parameter values. We use a measure of aggregation Π_*p*_ defined as

2.13 $$\Pi_{\;p}=1-\frac{{\left\langle \;p\right\rangle }^{2}}{\left\langle \;p^{2}\right\rangle },$$

where 〈 · 〉 represents a spatial average. Π_*p*_ ranges from 0 to 1 − 1/*N*^2^, where a value of 0 means there has been no aggregation (the distribution is homogeneous), and a value of 1 − 1/*N*^2^ means that all the plankton have aggregated to a single point in the grid, which has *N*^2^ mesh points.

We choose a realistic scenario where the phytoplankton are assumed to be neutrally buoyant so that *β*_*P*_ = 0, while the zooplankton are taken to be negatively buoyant with *β*_*Z*_ = −2.22 [12]. The size *L* of the (sub-mesoscale) eddies was taken to be 100 m across, and the maximum flow speed *U*_0_ was varied as a bifurcation parameter to investigate the response of the system to increasing spatial segregation caused by the Maxey and Riley term in equation (2.10). *D*_*P*_ and *D*_*Z*_ are set at a value of *D*_*P*_ = *D*_*Z*_ = 1.6 m^2^ s^−1^, in line with estimates of marine turbulent eddy diffusivity [26] for flow features of the order of a few kilometres. We chose this value for the diffusivity rather than the empirically derived value of 0.04 m^2^ s^−1^ suggested by Okubo [26], so that we could make a direct comparison with the results of Reigada *et al.* [12] as well as allowing rapid convergence of the numerical scheme. We have repeated the simulations with diffusivity *D*_*P*_ = *D*_*Z*_ = 0.04 m^2^ s^−1^, a reduction by a factor of 40. The results are qualitatively unchanged, and the bifurcation value for the flow parameter decreases by less than 10%. Importantly, this points to our mechanism of bloom formation being even more biophysically relevant than the results presented below.

Equations (2.4) and (2.5) were solved subject to periodic boundary conditions using a staggered mesh solver for the advection and diffusion components of the advection–diffusion–reaction equation, with an explicit Euler method used for the reaction terms. The numerical scheme was tested for convergence by repeating the simulations using a variety of grid spacing and time steps.

While the two-dimensional system provides archetypal solutions for an array of eddies, it is possible that the coupling between physical and ecological dynamics may be represented well in just one dimension, with a concomitant reduction in numerical complexity. Such a simplification would allow us to examine whether the observed two-dimensional dynamics are in any way attributed to the geometry associated with stagnation points and heteroclinic connections (corners) or stream-wise instabilities around the eddy. Axisymmetric eddies are an option but also require consideration of stagnation points at their outer boundaries if placed in a periodic array. Therefore, we avoid these topological issues and develop a simple approach to investigate the effect that drift into or out of an eddy has on the population dynamics. We model the concentration of plankton species across the diameter of a single eddy for a fixed value of *y* = *L*/4, so that the *x*-component *U*_1_ of the background flow given by (2.11) and (2.12) vanishes and the only remaining contribution to the zooplankton effective particle flow field in the *x*-direction comes from the Maxey and Riley drift term $\beta_{Z}[U\cdot \nabla ]U$ from (2.10). We can then calculate its *x*-component *V*_1*Z*_ for *y* = *L*/4 from (2.2). Recall that *P* is neutrally buoyant and so experiences no drift. Hence, (2.5) becomes

2.14 $$\frac{\partial Z}{\partial t}=-\frac{\partial }{\partial x}\left(V_{1Z}Z-D_{Z}\frac{\partial Z}{\partial x}\right)+f_{Z}(P,Z),$$

with no-flux boundary conditions at *x* = 0 and *L*/2, and similarly for ∂*P*/∂*t* in (2.4).

We use realistic values for the ecological parameters [14] and they can be found in table 1. The zooplankton death rate *μ* was chosen as a bifurcation parameter, taking values from 0.001 d^−1^ to 0.035 d^−1^. The first Hopf bifurcation occurs at *μ* = 0.0185 d^−1^ for the chosen parameter values, the transcritical bifurcation occurs at 0.0349 d^−1^, and so our range of values for *μ* ensures that we capture all qualitative behaviours of the excitable dynamical system.

**Table 1.** Ecological parameter values used in the simulations.

parameter	description	simulation values	unit
*r*	maximum phytoplankton growth rate	0.3	d^−1^
*K*	phytoplankton carrying capacity	108	μg N l^−1^
*R*_*m*_	maximum specific predation rate	0.7	d^−1^
*κ*	predation half-saturation constant	5.7	μg N l^−1^
*ε*	biomass conversion efficiency	0.05	—
*μ*	zooplankton mortality rate	0.001–0.035	d^−1^

In order to test the excitability of the system for different flow speeds, *U*_0_, we use the ideas of Truscott [27] and slowly change the value of *r* from an initial value *r*_0_. This acts as a perturbation to the system and allows us to find the critical value of d*r*/d*t* that results in the triggering of a bloom and leads to an excursion around phase space indicative of the system undergoing an excitation.

## 3. Results

All simulations were carried out using an initially homogeneous phytoplankton and zooplankton distribution corresponding to the ODE steady state (2.8), and iterated forward in time until all transients had decayed (1000 days) and the system exhibited stable limit cycle behaviour. The choice of *t* = 0 is arbitrary, and corresponds to the time of minimum spatially averaged phytoplankton population in the limit cycle.

**Figure 2.**
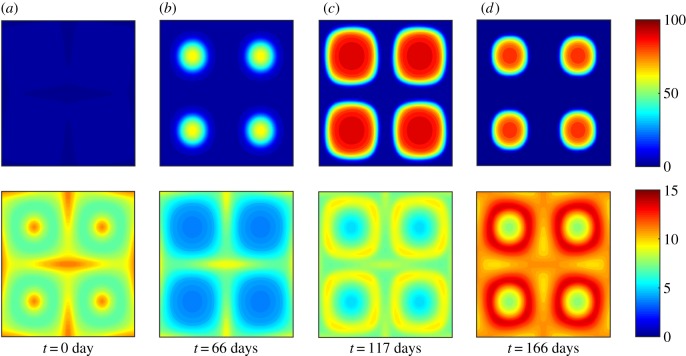
Large-scale blooms can be triggered via local flow effects. Spatio-temporal evolution of a plankton bloom triggered by spatial separation of predator and prey populations due to flow. An initially homogeneous distribution of zooplankton (bottom row) becomes concentrated in regions of low vorticity. This allows the phytoplankton population to escape grazing control in regions of high vorticity (top row), initiating a local bloom on an ecologically realistic time scale. (Online version in colour.)

Figure 2 shows snapshots of the model’s typical spatial output at four instants during the bloom cycle. At *t* = 0 days, the phytoplankton population *P* remains close to its homogeneous steady state, but spatial structure is apparent in the zooplankton population *Z*, with individuals advected away from the centre of the eddies. At *t* = 66 days, *Z* is sufficiently depleted within the eddies that *P* can increase in these regions due to the local removal of grazing control; a local bloom is initiated. By *t* = 117 days, the local *P* bloom has reached its peak and spreads diffusively towards the edges of the eddies. This gives *Z* an increased opportunity to consume its prey in regions of lower vorticity, leading to increased predator growth on the fringes of the circulations and a decrease in *P* back towards its minimum.

**Figure 3.**
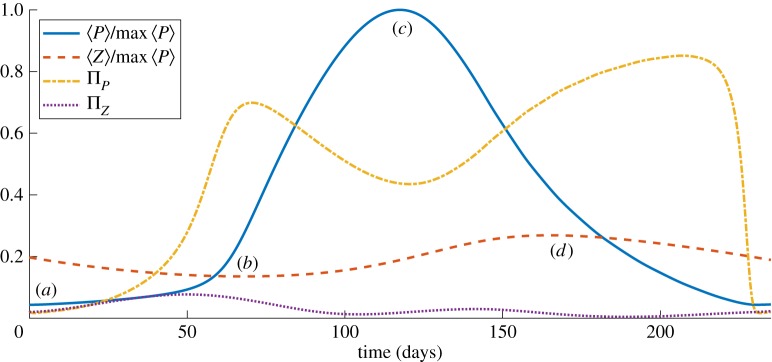
Evolution of spatial aggregation and population dynamics driven by flow. Dynamics of mean population size (blue for *P*, red for *Z*) and aggregation (yellow for *P*, purple for *Z*) of phytoplankton and zooplankton showing that the phytoplankton bloom occurs for a time of the order of weeks and is initiated shortly after zooplankton aggregation reaches a maximum. (Online version in colour.)

Figure 3 is an alternative depiction of these dynamics, detailing the temporal evolution of the mean population size (normalized by maximum *P* population) and aggregation measures of the *P* and *Z* populations, Π_*P*_ and Π_*Z*_, respectively, over a bloom cycle. At around *t* = 50 days zooplankton aggregation Π_*Z*_ reaches its maximum. As a consequence, the *P* population undergoes a rapid increase on a time scale of the order of days, indicating that the accumulation of zooplankton towards the edges of eddies provides enough space for the phytoplankton in the centre of the eddies to escape local grazing pressure. This leads to a decrease in *P* aggregation; phytoplankton spread diffusively across the eddy, and a local minimum in Π_*P*_ is reached soon after the maximum point of *P* at location (*c*). The zooplankton are then able to eat prey on the edges of the eddies and their population begins to rise before reaching a maximum at location (*d*). The bloom persists for a time of the order of months for the chosen parameter values.

**Figure 4.**
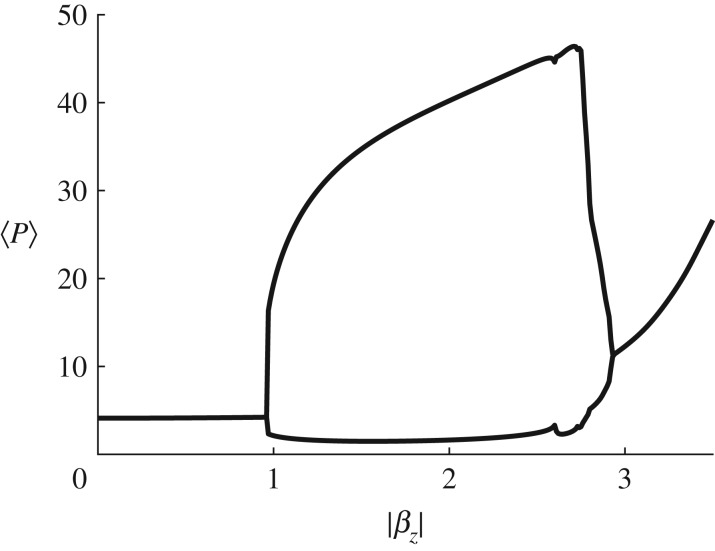
The Hopf bifurcation does not occur when inertial effects are too small. A bifurcation diagram showing the minimum and maximum spatially averaged phytoplankton density 〈*P*〉 for the two-dimensional system with inertia parameter |*β*_*Z*_| varying from 0 to 3.5, fixed maximum flow speed *U*_0_ = 3 m s^−1^ and zooplankton death rate *μ* = 0.012 d^−1^. (Online version in colour.)

**Figure 5.**
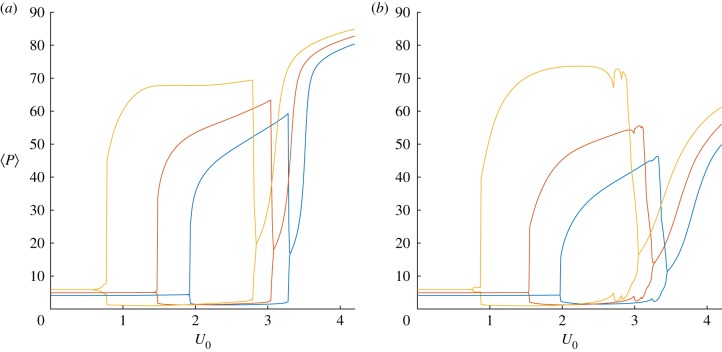
Flow-induced blooms exist across a large region of parameter space. Bifurcation diagrams showing the maximum and minimum spatially averaged phytoplankton density 〈*P*〉 for the one-dimensional (*a*) and two-dimensional (*b*) systems with maximum flow speed *U*_0_ varying from 0 to 4.2 m s^−1^ and zooplankton death rate *μ* = 0.012, 0.015 and 0.018 d^−1^ in blue, red and yellow, respectively. (Online version in colour.)

Figure 4 provides a bifurcation diagram with the inertia parameter *β*_*Z*_. Here, the flow speed *U*_0_ is set to 3 m s^− 1^, the zooplankton death rate *μ* is fixed at $0.012\;{\text{d}}^{-1}$ and the value of the inertia parameter *β*_*Z*_ is varied from −3.5 to 0. We plot the absolute value |*β*_*Z*_| to allow a direct comparison of the shape of the graph with those found in figure 5. The diagram demonstrates that there are no stable limit cycles for |*β*_*Z*_| below a value of approximately 1, at which a Hopf bifurcation occurs. A region of oscillatory solutions exists for|*β*_*Z*_| greater than this value but less than approximately 3. Beyond this value the spatially averaged inhomogeneous zooplankton population settles instead to a larger steady-state solution, which increases with the inertia parameter. These results allow us to establish a causal relationship between inertia and the initiation of oscillatory blooms in our model. It should, however, be noted that similar instabilities (in different physical regimes) can be induced in the absence of inertial effects, for example by a stretching flow with positive divergence [28] or through the interaction of Hopf and Turing mechanisms [29].

The bifurcation diagrams in figure 5 show that flow-induced blooms are a phenomenon which persists across a wide range of parameter values, and also that the essential features of the two-dimensional system (2.4), (2.5) are captured by the simpler model in one spatial dimension (2.14). The figures show the steady state of the system, or the maximum and minimum values of the oscillations in spatially averaged population density for regimes exhibiting oscillatory behaviour. As the flow magnitude *U*_0_ increases, we see that there exists a critical background flow speed above which an oscillatory domain of solutions (corresponding to cyclic blooms) is initiated via a Hopf bifurcation. The bifurcation point depends on both ecological and physical parameters, and the figures show that larger zooplankton death rates *μ* increase the likelihood that relatively small amounts of flow may induce blooms. It is interesting to note that all three of these *μ* values are beneath the Hopf bifurcation point of the underlying excitable dynamical system ($\mu =0.0185\;{\text{d}}^{-1}$), meaning that the physical flow effects are influencing the large-scale dynamics in all cases. Note that the critical flow speeds needed to induce oscillations (for each value of *μ*) are very similar in the one- and two-dimensional numerical models, indicating that the one-dimensional approximation is able to capture the behaviour of the full two-dimensional system for these parameter values. For larger flow speeds (*U*_0_ > 3.5 m s^−1^), the behaviour of the two systems starts to differ, with the one-dimensional system indicating a persistent bloom while the transition is more gradual in the spatially averaged output from the two-dimensional system. This is due to two-dimensional spatial effects, which can be understood by looking at the dynamics in more detail as explained below.

**Figure 6.**
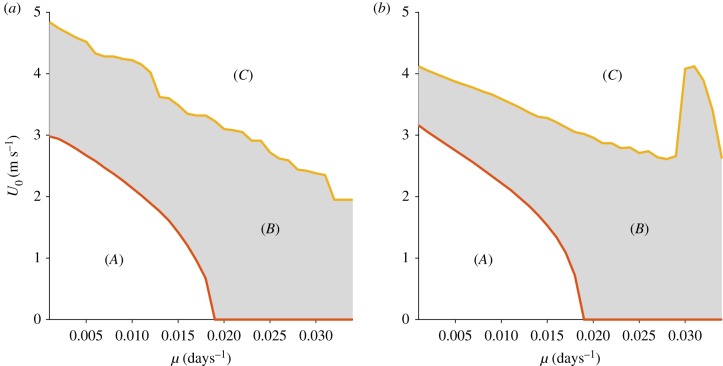
Regions of stable and oscillatory solutions, for a range of *Z* mortality *μ* and flow speed *U*_0_. (*a*) One-dimensional system. (*b*) Two-dimensional system. Oscillatory solutions, corresponding to flow-induced bloom dynamics, exist over a large area of parameter space (*b*). Region (*A*) corresponds to stable non-bloom dynamics for low flow speed (*U*_0_) and zooplankton mortality (*μ*) and region (*C*) to persistent blooms for large values of the same parameters. Critical combinations of flow and mortality are required to start a bloom. The bifurcation structure is complex close to the upper (yellow) boundary and while there is some numerical sensitivity there is convergence to a curve with geometrically interesting features. (Online version in colour.)

**Figure 7.**
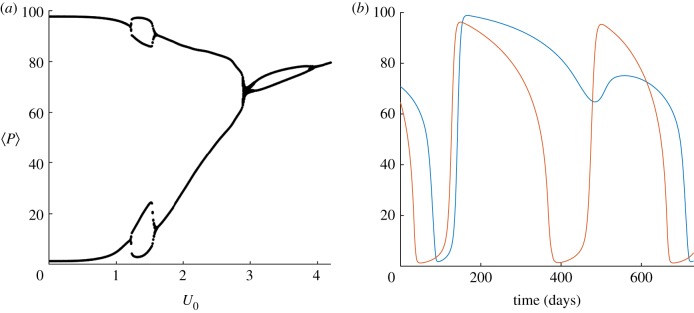
Local secondary oscillations in the two-dimensional model. (*a*) Bifurcation diagram showing spatially averaged phytoplankton concentration for $\mu =0.03\;{\text{d}}^{-1}$ and varying *U*_0_. (*b*) The mean phytoplankton concentration within a small box inside an eddy (blue), compared with that outside the eddy (red) for *U*_0_ = 1.3 m s^−1^; note the contrasting local period-2 oscillations, which account for the observed period doubling at the spatially averaged scale in (*a*). (Online version in colour.)

Figure 6 depicts a two-parameter bifurcation diagram of *U*_0_ against *μ* for both the one-dimensional and two-dimensional systems. The lower (red) lines indicate the critical parameter value pairs corresponding to the initiation of oscillatory solutions, while the upper (yellow) lines indicate parameter value pairs beyond which oscillations no longer can be found in the solutions. In both systems, region (*A*) is of a very similar shape and size, providing more evidence that the one-dimensional approximation is reasonable for small background flow speeds. However, differences appear for larger values of *μ* and *U*_0_ with an extra region of oscillatory solutions occurring in the two-dimensional system for *μ* > 0.03. Figure 7*a* helps to explain this extended oscillatory region, showing that the strict dichotomy between stable equilibrium and stable limit cycle regimes is not perfectly inherited from the non-spatial and one-dimensional systems. Instead, there are parameter choices containing additional small oscillatory modes at the spatially averaged scale. Figure 7*b* shows that these oscillations are caused by differences in local spatial dynamics. We plot the mean phytoplankton concentration within a small box inside an eddy (blue), and contrast this with the mean concentration outside the eddy (red) for a region of parameter space where secondary *P* − *Z* oscillations exist. Interestingly, while the *P* population outside the eddy has a regular oscillation and crashes approximately every 280 days, the population within the eddy crashes only every other cycle; the eddy provides some protection from grazing. These descriptions are valuable both in demonstrating the general predictive utility of the one-dimensional model and in illustrating the secondary local flow-induced structures which may arise.

**Figure 8.**
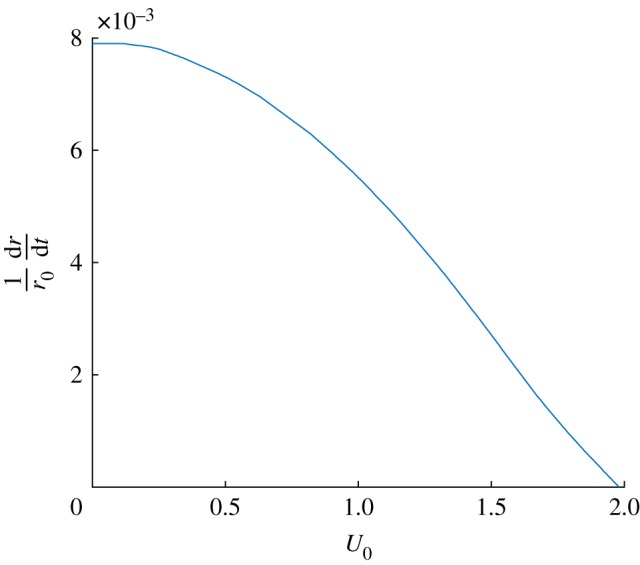
The perturbation threshold for excitable behaviour decreases with maximum flow speed. Plot showing the value of (1/*r*_0_)(d*r*/d*t*) against *U*_0_, with $\mu =0.012\;{\text{d}}^{-1}$, for values between *U*_0_ = 0 and 1.98 m s^−1^, the Hopf bifurcation point. (Online version in colour.)

As a measure of excitability, in figure 8 we plot the critical value of (1/*r*_0_)d*r*/d*t* required for a trajectory to take a large excursion around mean *P* − *Z* phase space rather than returning directly to the coexistence equilibrium point (2.8). This value is plotted against *U*_0_ with $\mu =0.012\;{\text{d}}^{-1}$. The curve is seen to meet the *x*-axis at around *U*_0_ = 1.98 m s^−1^, which corresponds to the lower Hopf bifurcation point in figure 5*b*. Therefore, even if flow speeds are not sufficient to cause the system to oscillate, an increase in flow speed can result in enhanced bloom excitability in the presence of an auxiliary environmental perturbation.

## 4. Discussion

We have shown that the inclusion of physical effects, such as small differences in inertia or buoyancy between predators and prey, can dramatically affect encounter rates between planktonic species, and that these changes can have consequences at ecological scales.

For illustration, the one-dimensional model indicates that the cross-stream velocity of a copepod of radius *a* = 5 mm of density *ρ*_*p*_ that is 10% more dense than water, *ρ*_*f*_, in an eddy of radius *L*/2 = 100 m with maximum flow velocity *U*_0_ = 1 m s^−1^ is given by $V=-(2/9)((\rho_{\;f}-\rho_{\;p})a^{2}U_{0}^{2}\pi /\eta L)\sin(4\pi x/L)\;{\text{m s}}^{-1}$, giving a maximum drift speed of 9 mm s^−1^. Even with a mean drift speed of 1 mm s^−1^ the organisms will migrate to the fringes of the eddy in a time of order 1 day. Over a time scale of several days, segregation between species and thus a significant reduction of grazing can occur. This has the effect of forcing trajectories in population dynamic phase space. If the underlying system is excitable then large excursions away from steady states are expected. Moreover, population dynamics where phytoplankton–zooplankton cycles are present (limit cycles or more complex attractors) in fully mixed systems can be quenched by inertia-induced drift (e.g. figure 5).

The numerical results show that blooms can be triggered by increased circulation flow speeds leading to greater spatial segregation between predator and prey. Hence, the flow itself can not only induce plankton bloom formation but can also qualitatively impact the population dynamics, shifting oscillatory dynamics to steady states and vice versa.

One criticism of the current approach is that the population dynamics depend only on local concentrations and not fluxes. Clearly, higher contact rates may increase grazing of phytoplankton by zooplankton, an effect that could be considered in future investigations, in line with Lewis *et al.* [30].

For mid-range flow speeds (typically, 1 − 3 m s^−1^ with our set of parameters), the inertial terms drive solutions away from steady states into oscillatory bloom solutions. Essentially, slightly dense zooplankton are gradually drawn out of eddies where there is a relatively low mean phytoplankton number density. The resultant reduction in grazing in the centre of the circulation reduces the constraint on phytoplankton growth and they are observed to bloom. However, large local gradients of phytoplankton concentration inevitably drive diffusion down the gradients. The zooplankton graze the phytoplankton at the edge of the eddies and grow in number themselves, generating diffusive fluxes of zooplankton that swamp inertial fluxes, and leading to consumption and repression of the eddy-focused bloom down to levels below the steady state. For the parameter ranges explored, any non-negative predator death rate (below the rate at which the coexistence equilibrium disappears) permits oscillatory solutions for some range of flow speeds.

Sufficiently large flow speeds (typically *U*_0_ > 3 m s^−1^) lead quickly to disaggregation of species, with a zone of overlap between *P* and *Z*. Oscillatory dynamics are lost and phytoplankton are seen to reach high concentration in the centre of eddies, bounded above by the carrying capacity *K*. The observed mean phytoplankton concentration reflects the increasing size of the zooplankton-absent zone with flow speed.

The ecological model presented herein is a simple and mathematically tractable way to capture the excitable plankton dynamics between two trophic levels, predator and prey. It is notable, however, that many HABs involve mixotrophic species [31]. For example, the bloom-forming dinoflagellate *Noctiluca scintillans* is a mixotrophic species which both feeds on phytoplankton and exploits the photosynthetic ability of ingested *Pedinomonas noctilucae* living in their vacuoles. Indeed, because the ingested microalgae may themselves be toxigenic, this mixotrophic relationship has been postulated as a mechanism which may increase HAB toxicity [32]. For species whose flow-related biophysical parameters are known, the methods of Hammer & Pitchford [33] can be adapted to quantify the joint role of mixotrophy and fluid motion in HAB formation, and will form a useful subject of future work.

The results in this paper are for a horizontal two-dimensional flow, and demonstrate that the interaction between physical and ecological systems gives rise to consequences unaccounted for by either system on its own. The model takes a simplified view of mixing by only including effective eddy diffusivity as a means for cells to spread out across the spatial domain, while the ability for cells to accumulate due to the Maxey and Riley effects is the cause of spatial segregation between predator and prey. However, much mixing occurs in the vertical direction. In order to consider the impact of vertical mixing one must also give careful consideration to sedimentation, light dependence and physical effects at the upper and lower boundaries. Behrenfeld & Boss [34] give a comprehensive overview of the effect of nutrient and light availability on phytoplankton biomass and how these change with mixed layer depth, building on the seminal work of Sverdrup [1].

At leading order one might assume that sedimenting organisms are spherical and that gravitational torques and biased swimming motion can be neglected. However, this is generally not the case. For instance, many plankton, such as diatoms, are markedly elongated and this can have a dramatic effect on sedimentation velocity [13]. Also, many species are bottom heavy or subject to sedimentary torques due to body asymmetry and swim in biased directions relative to gravity [6,35]. The growth of phytoplankton is very much dependent on the light availability, and the phytoplankton may themselves be phototactic [36]; models could include the well-known Lambert–Beer law for light attenuation and thus growth, and may also incorporate upward motion. Finally, there are different scenarios regarding the lower boundary condition: no-flux and no-slip conditions suggest a shallow sea whereas to model a mixed layer overlying deeper seas requires careful consideration of biomass loss and nutrient upwelling events [37]. All of these aspects merit further detailed study.

## Supplementary Material

Woodward et al. 1D Excitable Plankton Model Code

## Supplementary Material

Woodward et al. 2D Excitable Plankton Model Code

## Supplementary Material

Woodward et al. Graph Plotting Code

## Supplementary Material

Woodward et al. Graph Plotting Data

## Data accessibility

Model code files and data for plots are included in the electronic supplementary material.
